# Increased risk of venous thromboembolism in patients with acute leukaemia

**DOI:** 10.1038/sj.bjc.6602945

**Published:** 2006-01-17

**Authors:** M Mohren, I Markmann, K Jentsch-Ullrich, M Koenigsmann, G Lutze, A Franke

**Affiliations:** 1 Klinik für Hämatologie/Onkologie, Universität Magdeburg, Leipziger Str. 44, D-39120 Magdeburg, Germany; 2 Institut für Klinische Chemie, Universität Magdeburg, Leipziger Str. 44, D-39120 Magdeburg, Germany

**Keywords:** acute leukaemia, venous thromboembolisms, central venous catheters

## Abstract

Patients with malignancies have an increased risk for venous thromboembolisms (VTE), but data on patients with acute leukaemia are very limited so far. We found VTE in 12% of 455 patients with acute leukaemia, half of which occurred in association with central venous catheters, with equal risk of ALL and AML.

Venous thromboembolisms (VTE) may occur in patients with cancer and cause substantial morbidity and mortality. Since the first report back in 1868 (Trousseau, 1868), many publications have dealt with the association of thromboembolic and malignant diseases (Prandoni *et al*, 1992; Baron *et al*, 1998; Sorensen *et al*, 1998; Sutherland *et al*, 2002; Deitcher, 2003; Lee *et al*, 2003; Murchison *et al*, 2004; Otten *et al*, 2004). A recently published study reported VTE in 7.6% of 1041 patients with solid tumours (Sallah *et al*, 2002), whereas we found VTE in 7.7% of 1038 patients with malignant lymphoma with a higher incidence in high-grade than in low-grade lymphoma (Mohren *et al*, 2005). Data on patients with acute leukaemia have been scarce until very recently. However, an increased standardised incidence ratio of preceding VTE has been noticed in AML among other malignancies (White *et al*, 2005).

Otherwise frequent complications of acute leukaemia include thrombocytopenia-associated bleeding and granulocytopenia-related severe infection. Thus, thromboembolic complications have so far received little attention. As we observed coincidently VTE especially related to central venous catheters in a small number of consecutive leukaemia patients, we decided to initiate this retrospective study.

## PATIENTS AND METHODS

Medical records of all patients with acute leukaemia treated in our institution between January 1992 and April 2005 were reviewed. Patients with acute lymphoblastic and acute myeloid leukaemia as well as CML blast crisis (BC) according to the WHO 1997 criteria (Harris *et al*, 1999) were included in this study. Patients with myelodysplastic syndrome, myeloproliferative disease or CLL were excluded from the analysis. Venous thromboembolisms was confirmed either by venography or duplex ultrasound (deep vein thrombosis, central venous catheter-associated thrombosis), CT-scan or scintigraphy (pulmonary embolism) or by fundoscopy (central vein thrombosis). Superficial thrombophlebitis or central venous catheter occlusions without thrombosis were not counted as a VTE. Data were collected and analysed in a microsoft excel database.

## STATISTICAL ANALYSIS

*P*-values to show correlation of VTE with leukaemia lineage, patient age and gender were obtained using Fisher's exact test. All reported *P*-values are two-sided. As only one of 37 patients with blast crises had VTE, this patient group was not included in further correlation analysis.

## RESULTS

A total of 455 patients with acute leukaemia were eligible for analysis. In total, 248 patients (55%) were male, 207 were female (45%) and median age was 60 years. A total of 310 patients had AML (68%), 108 had ALL (24%) and 37 (8%) had BC (Table 1). Of the 310 patients with AML 89 (28.7%) had secondary leukaemia and only seven of the remaining 221 patients (3.2%) had promyelocytic leukaemia (PML) that is frequently associated with disseminated intravascular coagulation and/or hyperfibrinolysis. Among patients with PML, three had VTE: one patient had CVC-associated VTE after her third chemotherapy (cytarabine and idarubicine) course. She had been in complete haematological remission and was not taking ATRA at that time. The other two patients with PML had deep vein thrombosis during follow-up while still in complete remission.

There were 55 patients (12.1%) with at least one VTE. Venous thromboembolisms included central venous catheter-associated venous thrombosis in 27 patients (5.9%) and 28 non-central venous catheter-associated VTE (6.2%) (Table 2). The latter comprised deep vein thrombosis (*n*=20), upper extremity deep vein thrombosis (*n*=2), pulmonary embolism (*n*=5), thrombosis of the inferior caval vein (*n*=1) and central vein thrombosis (*n*=1). Only one patient had deep vein thrombosis on first presentation of ALL (lymphocytic blasts 45 G/l, platelets 265 G/l), whereas the great majority of VTE occurred during therapy (82%). The overall VTE rate was equal in ALL and AML (*P*=1.0). However, patients with ALL were less likely to develop central venous catheter-associated venous thrombosis than patients with AML, although the difference failed to reach statistical significance (*P*=0.119**)**. In two of 10 patients with ALL (20%) and non-central venous catheter-associated VTE, deep venous thrombosis occurred in association with the use of asparaginase. There was no difference in the VTE rate in male *vs* female patients with acute leukaemia (*P*=0.193) (Table 3), but patients younger than 60 years had a higher risk for VTE than patients of 60 years or more (*P*=0.014), attributed to a higher incidence of central venous catheter-associated venous thrombosis (*P*=0.003). There was no statistically significant difference in the rate of non-central venous catheter-associated VTE between both age groups (*P*=0.563) (Table 4). Only one patient with BC had a VTE.

## DISCUSSION

In this retrospective analysis, we found VTE in 12.1% of patients with acute leukaemia, a rate that is even higher than recently reported numbers from patients with other malignancies such as solid tumours or malignant lymphomas. No difference was found between AML and ALL. Central venous catheter-associated thrombosis were particularly common, accounting for half of all VTE in this cohort of leukaemia patients, whereas deep vein thrombosis and/or pulmonary embolisms were responsible for the other half of VTE. The rate of non-central venous catheter-associated thrombosis of 6.2% is very similar to those found in other malignancies (Sallah *et al*, 2002; Mohren *et al*, 2005).

The higher overall VTE rate in patients younger than 60 years in our study is due to the high incidence of central venous catheter-associated venous thrombosis in this patient group. This finding was made owing to the larger number of inserted central venous catheters in young patients, older patients often experiencing a very dismal prognosis and rather receiving palliative treatment rather than aggressive chemotherapy.

Only two very recently published studies have dealt with the association of VTE and acute leukaemia: in an observational cohort study of 379 acute leukaemia patients, 3.4% had VTE as a presenting manifestation and further 3.2% developed VTE within 6 months after diagnosis of leukaemia (de Stefano *et al*, 2005). Patients with ALL receiving L-asparaginase were at particularly high risk. A retrospective Austrian study looked at the incidence of VTE in close temporal relationship to the onset of acute leukaemia in 719 patients and found a VTE rate of 2.1% with equal risk in AML and ALL (Ziegler *et al*, 2005). Both studies found a high rate of VTE at first presentation in patients with PML, an AML subtype that is frequently associated with either bleeding or thrombosis (Dally *et al*, 2005). In contrast to these findings, none of our patients with this AML subtype had VTE on diagnosis of leukaemia. However, three of seven patients with PML (42%) in our cohort developed VTE later during the course, when leukaemia was no longer present, rather a coincidental finding than a true association. Surprisingly, only 3.2% of patients in our cohort had PML, a lower than expected number.

In comparison to both previous studies, we observed a higher overall rate of VTE. This may be partially due to the longer observation period in our retrospective study. Ziegler *et al* investigated acute leukaemia associated VTE in close temporal relationship with diagnosis of the leukaemia only and de Stefano *et al* reported an observation period of 6 months after disease onset. However, since half of the VTE occurring in our patient cohort were associated with the use of central venous catheters, this complication is most likely responsible for the substantially higher VTE rate observed in our analysis. Our reported rate of non-central venous catheter-associated thrombosis of 6.2% is comparable to the VTE rate observed by de Stefano *et al*, although we failed to find a statistically significant difference between AML and ALL.

A high rate of central venous catheter-associated thrombosis of 12–15% in patients with haematological malignancies including acute leukaemia has been reported in two previous studies from Italy, and these numbers are similar to those reported from non-thrombocytopenic patients with central venous catheter-associated thrombosis (Cortelezzi *et al*, 2003, 2005). Since the authors counted superficial thrombophlebitis as a venous thromboembolic event and not all our patients had a central venous catheter inserted, no direct comparison of the central venous catheter-associated thrombosis rates observed in the studies by Cortelezzi *et al* with our data is possible. However, these findings are supporting evidence that central venous catheter-associated thrombosis is responsible for a considerable number of thromboses in patients with acute leukaemia.

We conclude that VTE are frequent in patients with acute leukaemia; thus, prophylactic measures even in thrombocytopenic patients need to be established, particularly in patients with central venous catheters.

## Figures and Tables

**Table 1:** Patients with acute leukaemia: total number and distribution according to leukaemia type and patient gender

	**All patients**		**Male patients**		**Female patients**	
	**(*n*)**	**(%)**	**(*n*)**	**(%)**	**(*n*)**	**(%)**
Acute leukaemia	455	100	248	54.5	207	45.5
AML	310	68.1	160	51.6	150	48.3
ALL	108	23.7	65	60.1	43	39.8
BC	37	8.1	23	62.1	14	37.8

**Table 2:** Patients with acute leukaemia (AL) and VTE: analysis according to leukaemia type and presence of a central venous catheter (CVC)

	**Total VTE**		**CVC-associated VTE**		**Non-CVC-associated VTE**	
	**(*n*)**	**(%)**	**(*n*)**	**(%)**	**(*n*)**	**(%)**
AL	55	12.1	27	5.9	28	6.2
AML	40	12.9	23	7.4	17	5.5
ALL	14	13.0	4	3.7	10	9.3
BC	1	2.7	0	—	1	2.7

**Table 3:** VTE in patients with AL according to age and gender

	**VTE (*n*)**	**VTE (%)**
Age⩾60 Jahre (*n*=229)	19	8.3
Age <60 Jahre (*n*=226)	26	15.9
Male (*n*=248)	25	10.1
Female (*n*=207)	30	14.5

**Table 4:** Central venous catheter (CVC)-associated and non-CVC-associated VTE in patients with acute leukaemia according to age

	**Age⩾60 years**		**Age<60 years**	
	***n***	**%**	***n***	**%**
CVC-associated VTE	6	2.6	21	9.3
Non-CVC-associated VTE	13	5.7	15	6.6
